# Case Report: Recurrent psychotic episodes in the setting of non-COVID-19 upper respiratory viral infection

**DOI:** 10.3389/fpsyt.2025.1546501

**Published:** 2025-03-07

**Authors:** Zahraa Atoui, Yi Ting Liu, Andrew Lee, Caroline Nardi, Erin Seery, McLeod Frampton Gwynette

**Affiliations:** Department of Psychiatry and Behavioral Sciences, Medical University of South Carolina, Charleston, SC, United States

**Keywords:** psychotic spectrum disorder, psychotic disorder due to another medical condition, upper respiratory viral infections, viral hypothesis for schizophrenia, case report

## Abstract

The viral hypothesis of schizophrenia suggests a link between viral respiratory infections and the development of psychotic symptoms. There have been several cases reporting development of new-onset psychosis after upper respiratory illnesses, including influenza and more recently coronavirus disease-2019 (COVID-19). Here, we present a case of a previously healthy African American female with no history of mental illness who developed psychotic symptoms following an upper respiratory illness in three distinct episodes at the ages of 16, 18, and 21. The patient had extensive medical workup during these episodes which did not identify an infectious source despite clear signs and symptoms of infection. Her psychiatric symptoms included disorganization, paranoia, response to internal stimuli and one episode with catatonic features. These symptoms improved with initiating antipsychotic treatment, and complete resolution was achieved within a couple months in the outpatient setting. In this report, we detail the disease course, medical workup, and selection of psychotropic treatments. This case highlights the challenges with identifying the diagnosis, completing appropriate workup, and selecting the best management for patients with similar presentations. It further demonstrates the connection between viral illnesses and development of psychosis and underscores the importance of further research to better understand patients with similar presentations.

## 1 Introduction

The viral hypothesis of schizophrenia has existed for decades, with increasing research and published case reports supporting this phenomenon. Since the German influenza pandemic in the 1920s when Karl Menninger linked the Spanish flu to psychosis (1), observations have been made connecting viral respiratory infections to neuropsychiatric diseases. More recently, viral infections such as influenza A virus subtype H1N1 (H1N1) and non-COVID-19 human coronavirus have been linked to psychosis (2, 3), mania (4), and depression (5).

Various mechanisms have been proposed to explain the connection between viral infections and psychosis. Immune activation has been shown to play a role in schizophrenia, with studies implicating the body’s immune-mediated response to infection, in addition to the presence and activity of the virus, in the development of psychiatric symptoms. Immune-mediated effects include elevated pro-inflammatory cytokines in the blood and cerebrospinal fluid (CSF) (6), complement proteins in the blood (7), and microglial activation (8). Maternal infections have also been implicated in the offspring’s development of psychosis (9) through neural damage in the developing brain caused by increased maternal immune and inflammatory responses (10, 11). Stress is another factor that can contribute to developing psychosis through the overactivation of the hypothalamic-pituitary-adrenal axis following viral infection (12).

With the COVID-19 pandemic in 2019, there was a rise in the number of studies reporting new-onset psychosis following infection (13–15). Observational studies estimated the incidence of psychosis following viral illness at 0.9%-4% (16), which is significantly higher than the general population’s incidence of 26.6 per 100,000 person-years (17). The exact mechanism behind this increased incidence remains largely unclear, and further research is needed to clarify the underlying pathways and contributing factors.

**Figure 1:**
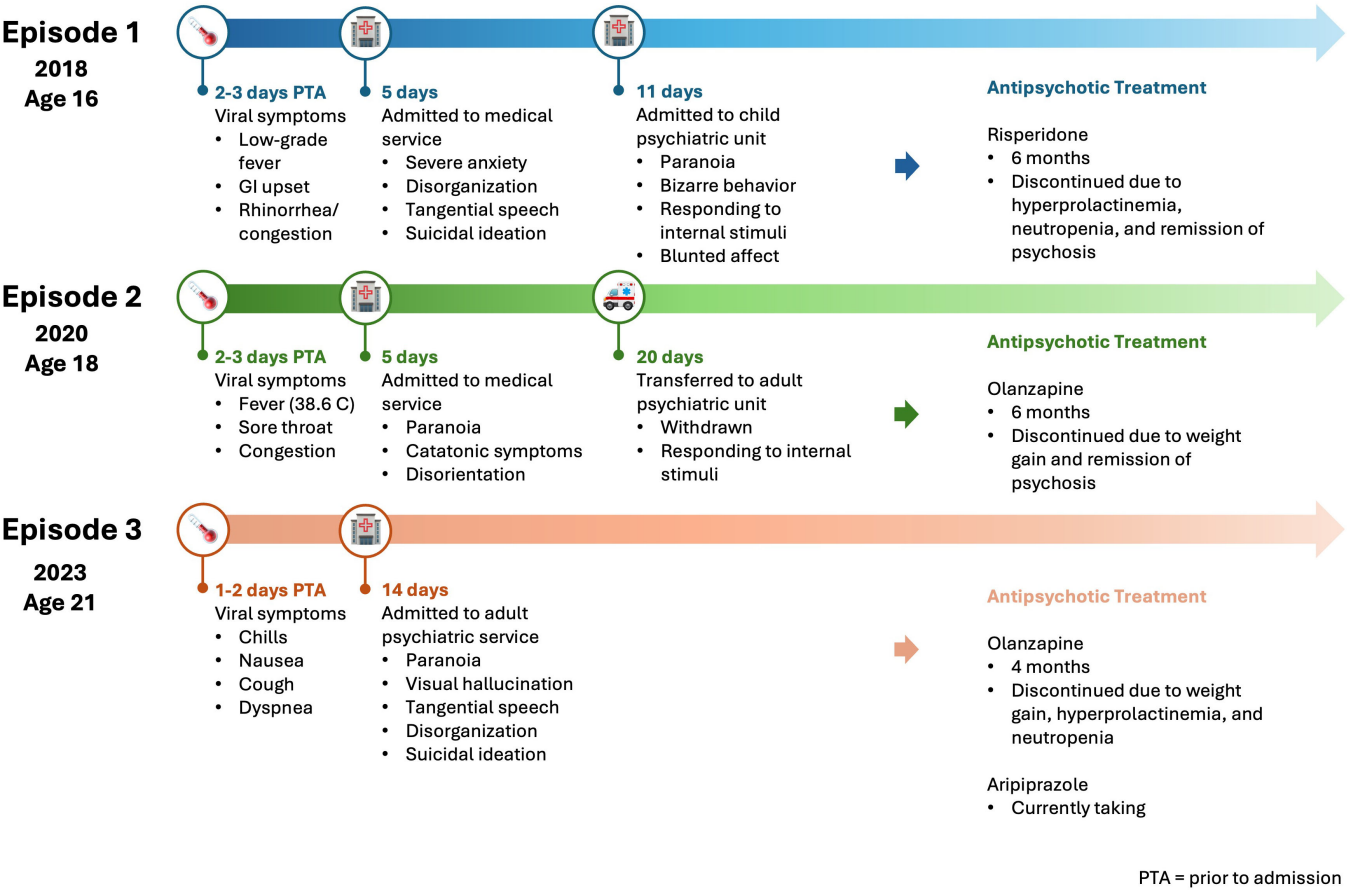
Timeline showing symptoms, transition of care, and treatment for each episode.

While many case reports link viral infections to psychosis temporally, recurrent psychotic episodes following viral infection have not been well-documented. Here, we present a patient who has had 3 brief psychotic episodes (**Figure 1** *showing the timeline and treatment*) in the setting of a non-COVID-19 viral infection, precipitating inpatient medical and psychiatric admissions for thorough work-up and treatment.

## 2 Case description

The patient is presently a 22-year-old African American female. Prior to her initial presentation, the patient had no past developmental, medical, or psychiatric history. Her family history is largely unremarkable except for a maternal half-brother with schizophrenia. The patient experienced three psychotic episodes, most recently in 2023. Medical work-up at each presentation was unrevealing, aside from clinical symptoms of upper respiratory infection.

### 2.1 First episode

The patient was 16 years old at the time of her first psychotic episode. She presented with acute anxiety, disorganization, agitation, and suicidal ideation in setting of rhinorrhea, congestion, gastrointestinal upset, and reported low-grade fever for several days. She was afebrile on presentation but was intermittently tachycardic and hypertensive. Vital sign instability improved upon administration of lorazepam as needed to target anxiety and agitation. Given the acute onset and symptom severity, she was medically admitted to the general pediatric unit for further work-up, with a leading differential diagnosis of anti-N-methyl-D-aspartate (anti-NMDA) receptor encephalitis. Basic work-up, including complete blood count (CBC), complete metabolic panel, urine studies, CSF studies, and imaging were all unremarkable (**Table 1**). The patient continued to respond to internal stimuli and exhibit paranoia throughout her 5-day stay. She was discharged home and then readmitted to the psychiatric service for worsening psychotic symptoms. Risperidone was consequently initiated to target symptoms of extreme paranoia, echolalia, bizarre behavior, and intermittently refusing to eat and drink. The patient was also on lorazepam 1 mg twice daily to help with anxiety. Symptoms improved with the initiation of 1 mg nightly of risperidone, though not completely resolved. She was discharged after 10 days on these medications and followed on an outpatient basis, where her risperidone was increased to 3 mg nightly while lorazepam tapered off within 6 weeks. Symptoms gradually improved and full resolution was achieved within 4 months. Unfortunately, the patient experienced symptomatic hyperprolactinemia (91 ng/ml) with irregular periods, and neutropenia (absolute neutrophil count (ANC)=0.98K/CUMM) during her treatment. Outpatient Hematology was consulted for the latter, which was thought to be a combination of benign ethnic neutropenia (BEN) and risperidone side effect at the time. With symptom resolution, the patient and her parents opted for cautious monitoring off antipsychotic medication. Risperidone was consequently tapered and discontinued after six months of treatment. The patient remained stable off risperidone for 18 months.

**Table 1:** Vital signs, laboratory studies, imaging, and special procedures performed for each episode of care.

Type of study	Results (Episode 1)	Results (Episode 2)	Results (Episode 3)
Vital signs	BP up to 150/99, HR up to 140, T up to 38.3°c	BP up to 170/106, HR up to 159, T 38.6°c	BP up to 135/94, HR up to 147, T up to 37.8°c
Basic blood and serum studies	WBC 5.83 K/CUMM (4.8-10.8), RBC 4.39 M/CUMM (4.2-5.4), Hemoglobin 12.5 GMS/dL (12-16), Hematocrit 37.9% (37-47), Platelet count 304 K/CUMM (140-440), Neutrophils 66.7% (45-70%), Lymphocytes 21.6% (20-45%), PT 14.2 sec (12-15.1), INR 1.05, aPTT 31.9 sec (23.3-35.7). Sodium 139.0 mmol/L (135-145), **Potassium 3.2 mmol/L** (3.5-5.1), Chloride 105.0 mmol/L (98-108), arterial Total CO2 22 mmol/L (20-28), Glucose 97 mg/dL (70-100), **BUN 5.0 mg/dL** (7.3-19), Creatinine 0.7 mg/dL (0.5-1), Calcium 9.5 mg/dL (9.2-10.5), Total Bilirubin 0.6 mg/dL (0.2-1.2), AST 14.0 U/L (13-26), ALT 12.0 U/L (8-22), Alkaline phosphatase 66.0 U/L (54-128), Albumin 4.0 G/dL (3.5-4.9), Total Protein 8.0 G/dL (6.5-8.1). Cholesterol 146.0 mg/dL (112-208), LDL 93 mg/dL (<=100), HDL 46 mg/dL (40-59), Triglycerides 35.0 mg/dL (<=150). **25-Hydroxy D Total 4.7 ng/mL** (normal range 25-80 ng/mL). TSH 2.50 mIU/L (0.47-3.63), Free T3 2.7 pg/mL (2.3-3.7), **Free T4 1.40 ng/dL** (0.67-1.22). Complement C3 101.1 mg/dL (83-152), Complement C4 33.1 mg/dL (13-37). Serum toxicology: Arsenic <10 ug/L (0-13), mercury <3 ug/L (0-10). Ammonia 45.2 umol/L (18-72). CRP <0.02 mg/dL, ESR 6 mm/hr (0-20)	WBC 5.6 K/CUMM (4.8-10.8), RBC 4.48 M/CUMM (4.2-5.4), Hemoglobin 12.9 GMS/dL (12-16), Hematocrit 38.7% (37-47), Platelet count 255 K/CUMM (140-440), **Neutrophils 71.1%** (45-70%), **Lymphocytes 18.2%** (20-45%). Sodium 139.0 mmol/L (135-145), **Potassium 3.3 mmol/L** (3.5-5.1), Chloride 106.0 mmol/L (98-108), arterial Total CO2 21 mmol/L (20-28), **Glucose 146 mg/dL** (70-100), **BUN 5.0 mg/dL** (7.3-19), Creatinine 0.7 mg/dL (0.5-1), Calcium 9.7 mg/dL (9.2-10.5), Magnesium 1.7 mg/dL (1.6-2.6), Phosphorus 3.1 mg/dL (2.9-5), Total Bilirubin 0.7 mg/dL (0.2-1.2), AST 13.0 U/L (13-26), ALT 10.0 U/L (8-22), Alkaline phosphatase 49.0 U/L (54-128), Albumin 3.9 G/dL (3.5-4.9), Total Protein 7.5 G/dL (6.5-8.1). TSH 2.06 mIU/L (0.47-3.63), Thyroxine T4 10.4 mcg/dL (5.5-13). Procalcitonin <0.05 ng/mL (<= 0.10). Salicylate level <5.0 mg/dL (5-20). Ethyl alcohol <10.0 mg/dL (0-10). Creatine Kinase 181 U/L (20-190). Folate 14.4 ng/mL (7-31.4). CRP 0.08 mg/dL (0-1), ESR 8 mm/hr (0-20).	WBC 6.63 K/CUMM (4.8-10.8), **RBC 4.17 M/CUMM** (4.2-5.4), Hemoglobin 12.1 GMS/dL (12-16), Hematocrit 36.9% (37-47), Platelet count 264 K/CUMM (140-440), Neutrophils 66.7% (45-70%), Lymphocytes 24.9% (20-45%). Sodium 139.0 mmol/L (135-145), **Potassium 2.9 mmol/L** (3.5-5.1), Chloride 106.0 mmol/L (98-108), CO2 22 mmol/L (20-28), Glucose 96 mg/dL (70-100), BUN 10 mg/dL (7.3-19), Creatinine 0.9 mg/dL (0.5-1), Calcium 9.2 mg/dL (9.2-10.5). **Creatine Kinase 210 U/L** (20-190). Cholesterol 145.0 mg/dL (112-208), LDL 82 mg/dL (<=100), HDL 53 mg/dL (40-59), Triglycerides 50.0 mg/dL (<=150). Lactate 1.2 mmol/L (0.5-1.6). Ethanol level <10 mg/dL (0-10)
Viral tests		Nasopharyngeal swab negative for: SARS Coronavirus 2 (COVID-19), Adenovirus PCR, Coronavirus 229E, Coronavirus HKU1, Coronavirus NL63, Coronavirus OC43, Influenza A, Influenza B, Parainfluenza Type 1, Parainfluenza Type 2, Parainfluenza Type 3, Parainfluenza Type 4, RSV, Rhinovirus/Enterovirus.	Nasopharyngeal swab negative for: SARS Coronavirus 2 (COVID-19), Adenovirus PCR, Coronavirus 229E, Coronavirus HKU1, Coronavirus NL63, Coronavirus OC43, Influenza A, Influenza B, Parainfluenza Type 1, Parainfluenza Type 2, Parainfluenza Type 3, Parainfluenza Type 4, Metapneumovirus, RSV, Rhinovirus/Enterovirus.
Bacterial tests		Blood culture with no bacteria or yeast isolated at 121 hours. Nasopharyngeal swab negative for Chlamydophila pneumoniae, Bordetella pertussis PCR, Mycoplasma pneumoniae PCR.	Nasopharyngeal swab negative for Chlamydophila pneumoniae, Bordetella pertussis PCR, Bordetella parapertussis, Mycoplasma pneumoniae PCR.
Urine Studies	Urine pregnancy test negative. Urinalysis negative for blood, nitrite, leukocyte esterase, bilirubin. **Protein 30 mg/dL** (<=10), **Ketones 20 mg/dL (**<=10), RBC <1/HPF (0-3), WBC 3/HPF (0-3), urobilinogen <2.0 mg/dL (<=2.0), **moderate mucus**, **few bacteria. ** Urine toxicology negative for amphetamine, barbiturates, benzodiazepines, cannabinoids, cocaine, phencyclidine, methadone, opiates, oxycodone/oxymorphone.	Urine pregnancy test negative. Urinalysis negative for blood, nitrite, glucose, leukocyte esterase, and bilirubin. **Protein 30 mg/dL** (<=10), **Ketones 20 mg/dL (**<=10), RBC 1/HPF (0-3), WBC 1/HPF (0-3), urobilinogen <2.0 mg/dL (<=2.0), **moderate mucus**, **few bacteria.** Uroporphyrin 0 (0-4). Urine toxicology negative for amphetamine, barbiturates, benzodiazepines, cannabinoids, cocaine, phencyclidine, methadone, opiates, oxycodone/oxymorphone.	Urine pregnancy test negative. Urinalysis negative for blood, nitrite, leukocyte esterase, and bilirubin. **Ketones 20 mg/dL (**<=10), RBC <1/HPF (0-3), WBC 1/HPF (0-3), urobilinogen <2.0 mg/dL (<=2.0), **few mucus**, **few bacteria.** Urine toxicology negative for amphetamine, barbiturates, benzodiazepines, cannabinoids, cocaine, phencyclidine, methadone, opiates, oxycodone/oxymorphone.
CSF studies	Glucose 64 mg/dL (40-70), Protein 16.8 mg/dL (15-45), WBC <1/CUMM, **RBC 5/CUMM** (0-1). Oligoclonal bands negative, IgG 1.4 mg/dL (0-6), Albumin 9 mg/dL (0-35). CSF autoimmune encephalopathy panel negative for NMDA Receptor Ab, VGKC-complex Ab, LGI1-IgG, CASPR2-IgG, GAD65 Ab, GABA-B-R Ab, AMPA-R AB, ANNA-1, ANNA-2, ANNA-3, PCA-1, PCA-2, PCA-Tr, Amphiphysin Ab, CRMP-5-IgG.	**Glucose 73 mg/dL** (40-70), **Protein 14.5 mg/dL** (15-45), WBC 1/CUMM, **RBC 3/CUMM** (0-1). CSF negative for: Enterovirus, HSV Type 1, HSV Type 2, HHV-6, Human Metapneumovirus. N-Methyl-D-Aspartate (NMDA) Receptor Antibody <1:1 (reference range <1:1). CSF culture with no microorganisms isolated.	
Serum antibody studies	Antinuclear antibodies (ANA) negative, Double strand DNA Antibody 2.0 IU/mL (<=9.9), Smith antibodies <0.2 AI (<=0.9), Thyroid peroxidase antibodies <15 IU/mL (<=34.9), TSH receptor antibody (TRAB) <0.9 IU/L (<= 1.75 IU/L). Syphilis AB IgG <0.2 AI (<=0.8). Nonreactive HIV-1 and HIV-2 antibodies	Antistreptolysin O 45 IU/mL (0-530). Nonreactive HIV-1 and HIV-2 antibodies. Serum Autoimmune Encephalopathy panel negative for AchR Ganglionic Neuronal Ab, AMPA-R Ab, Amphiphysin Ab, AGNA-1, ANNA-1, ANNA-2, ANNA-3, CASPR2-IgG, CRMP-5-IgG, DPPX Ab, GABA-B-R AB, GAD65 Ab, GFAP, IgLON5, LGI1-IgG, mGluR1 Ab, NIF, NMDA-R Ab, N-Type Calcium Channel Ab, P/Q-Type Calcium Channel Ab, PCA-1, PCA-2, PCA-Tr.	
Imaging	MRI brain with/without contrast: small focus of T2 hyperintensity within the left paramedian frontal lobe vertex, nonspecific, and 2 small foci of susceptibility artifact within the posterior left temporal lobe, may represent chronic microhemorrhage. Findings reflecting prior trauma. Pelvic ultrasound is significant for an indistinct area of increased echogenicity in the right adnexa which may represent bowel gas, however images are limited. Renal ultrasound is unremarkable. MRI Pelvis with/without contrast: normal with no adnexal mass.	Chest X-ray AP Portable with no focal airspace disease. CT head without contrast with no acute intracranial abnormality. Abdominal ultrasound with increased cortical echogenicity bilaterally, which can be seen in medical renal disease. Pelvic ultrasound with 1.5 cm right simple ovarian cyst, otherwise normal.	
Other studies		Routine EEG normal for age in the awake state.	

Bolded values indicate results that fall outside the normal reference range for the lab.

### 2.2 Second episode

The patient was 18 years old during her second episode in 2020. She developed a similar clinical picture including disorganized behavior, paranoia, and hallucinations in the setting of another viral upper respiratory illness. She had a sibling who tested positive for a streptococcal throat infection earlier that week. The patient reported sore throat, congestion, and an episode of nocturia. She had altered mental status and fever on admission to the medical unit. The patient underwent an extensive medical work-up, which again was unrevealing (see **Table 1**). She continued to exhibit psychotic symptoms, with new-onset odd posturing, waxy rigidity, and autonomic instability, which warranted her transfer to the psychiatric inpatient unit for the management of psychosis with catatonic features. Lorazepam was started at 1.5 mg four times daily and titrated gradually over 10 days to 5 mg four times daily, which slightly improved catatonic symptoms. Given previous side effects with risperidone, olanzapine was started at 2.5 mg daily, increased to 5 mg on the third day, 10 mg on day 5, 15 mg on day 11, and eventually titrated to 20 mg daily on day 14. The patient’s catatonic and psychotic symptoms improved, allowing for the reduction of lorazepam to 3 mg three times daily by the time of discharge with the plan to complete the taper on an outpatient basis. The patient’s symptoms completely resolved within two months of discharge. While taking olanzapine, the patient experienced severe side effects, including excessive sedation, a 40 lb weight gain, irregular periods (prolactin was normal at 9.4 ng/mL), and neutropenia (ANC=1.47K/CUMM). Olanzapine was therefore decreased to 10 mg daily 2 weeks after discharge, and then 5 mg 2-3 months later, and metformin was initiated. However, this was ineffective in mitigating side effects. Given complete resolution of psychotic symptoms, olanzapine was eventually discontinued 6 months post-treatment with a plan to cautiously monitor. The patient’s menstrual cycles stabilized, her sedation improved, and she lost 12 lb within four months following olanzapine discontinuation.

### 2.3 Third episode

The patient was 21 years old during her third psychotic episode in 2023. She presented again with insomnia, disorganization, agitation, and was responding to internal stimuli in the setting of viral symptoms, including cough, chills, dyspnea, and subjective fevers at home. Given previous unremarkable work-up during her prior episodes, the focus was on promptly addressing her symptomsafter obtaining a basic work-up in the emergency department (**Table 1**), including a negative COVID-19 test. The patient was then admitted to the psychiatric service. No catatonic symptoms were noted during this admission. Olanzapine was restarted at 5 mg nightly and titrated to 10 mg twice daily byday 7 without significant side effects during her inpatient stay. The patient’s symptoms improved significantly, and she was discharged after 14 days with outpatient follow-up. She had complete resolution of symptoms within one month of olanzapine initiation.

### 2.4 Follow-up care

The patient continues to receive care in our outpatient general adult psychiatry clinic to date. On follow up, the treatment team discussed the risks and benefits of long-term antipsychotic maintenance treatment, given her repeated episodes of psychosis. The patient agreed to continue antipsychotic treatment indefinitely. Olanzapine, however, caused significant fatigue, irregular menses, and weight gain of 20 lb over 4 months, with body mass index (BMI) increasing from 25.2 to 29 kg/m^2^. Lab work-up showed symptomatic hyperprolactinemia (117.8 ng/ml) and neutropenia (ANC=1.52K/CUMM). Given these side effects, we cross-titrated to aripiprazole over a 4-week period to a final dose of 10 mg daily. Repeat prolactin and ANC levels were within normal ranges (7.4 ng/ml and 2.60 K/CUMM respectively).

The patient has maintained stability on aripiprazole 10 mg daily since 2023. Despite having three severe psychotic episodes, she persistently returned to her full baseline functioning between episodes and is currently resuming her graduate studies to become a healthcare professional at a prestigious university. Of note, the patient had a COVID-19 infection in July 2024 while on aripiprazole treatment (confirmed via home test kit), with symptoms including nausea, vomiting, cough, rhinorrhea and myalgia, but had no re-emergence of psychotic symptoms. The patient reported receiving four COVID-19 vaccinations, most recently in November 2023. Nonetheless, this was the first significant respiratory illness this patient experienced while on antipsychotic maintenance treatment. Current major challenges include weight gain (BMI=31.5 kg/m^2^). This is being addressed through lifestyle modifications and metformin.

## 3 Diagnostic assessment

On initial presentation, the patient exhibited disorganized thoughts and behaviors in the setting of an upper respiratory illness. An extensive medical work-up was performed, with broad differential diagnoses including central nervous system (CNS) infection, anti-NMDA receptor encephalitis, and lupus cerebritis. Neurology, nephrology, rheumatology, and psychiatry services were consulted. Her neurological exam was benign. Brain magnetic resonance imaging (MRI) showed nonspecific changes reflecting prior trauma and was deemed unremarkable by neurology. Abdominal and pelvic imaging ruled out ovarian tumor, commonly associated with anti-NMDA receptor encephalitis (18). Lumbar puncture, CSF studies, oligoclonal bands, and CSF encephalopathy panel were all unremarkable. No seizure history was reported, nor an activity observed. Consequently, anti-NMDA receptor encephalitis and CNS infection were ruled out. Serum antibody markers were not concerning for lupus cerebritis. With a largely negative medical work-up, the patient was then treated with a second-generation antipsychotic on the psychiatric service. She returned to baseline within 4 months, and antipsychotic treatment was discontinued after 6 months. The second episode was again significant for upper respiratory illness followed by acute decompensation in behavior and psychiatric symptoms. Although her brother tested positive for strep infection that week, the patient’s full infectious and neurological work-up was again negative, including COVID-19, and the patient was transferred to the psychiatric service for treatment of psychosis with catatonic features. With lorazepam titration and re-initiation of an antipsychotic, she returned to baseline within two months. The antipsychotic was again discontinued after 6 months of treatment due to symptom resolution and poor tolerability. On her third presentation, the patient underwent a basic medical and infectious work-up which was unrevealing and then was treated on the psychiatric service. Similarly, she had a brisk return to baseline function with treatment.

The thorough medical work-up performed during her hospitalizations was unsuccessful in isolating a viral origin for her psychiatric symptoms. Common causes of delirium and encephalopathy were ruled out. Nonetheless, upper respiratory symptoms were evident directly before her psychotic symptom onset. Given the atypical age of onset, brevity of episodes, temporal relationship to viral infections, lack of gradual deterioration, and rapid return to a high functional baseline with treatment, we believe that this patient’s psychosis is likely secondary to a medical condition and precipitated by an immune-mediated response to a viral respiratory illness.

## 4 Discussion

The presented case has several notable features. First, the patient had recurrent psychotic episodes, each preceded by upper respiratory viral infection and vital sign instability. There have been some reported cases of adolescents developing single, brief psychotic episodes after a viral illness. According to a chart review by Tzang et al., 21 out of 60 patients with a psychosis-related diagnosis in a Taiwanese hospital system were noted to develop psychosis after an upper respiratory infection and required antipsychotic treatment. Most notably, researchers specified that after the resolution of the viral infection and transient psychotic symptoms, these patients did not return for further treatment of psychosis and did not have subsequent psychotic episodes (19). In this case, the patient did have recurrent psychotic episodes, all in the context of viral illness and systemic symptoms. The episodes started in adolescence and continued into young adulthood. Unfortunately, a specific viral etiology was not identified; it is unknown whether these episodes were elicited from the same strain.

Second, this case is distinct as the patient did not receive steroids during her course of treatment. Many reported cases of psychosis linked to a viral illness, particularly COVID-19, were confounded by steroids (20, 21). The occurrence of psychiatric symptoms, including psychotic and mood symptoms, with steroid-use has been well documented (22). Additionally, her presentation was not complicated by alcohol or other substance use, demonstrated by negative urine drug screens and history provided by the patient and her family on all three admissions.

Lastly, the patient had an episode of psychosis accompanied by catatonic-like symptoms. Catatonia is most commonly of psychiatric etiology, however about 20% of catatonic cases are attributable to medical causes, with over two-thirds due to CNS-specific conditions such as encephalitis, neural injury, and neurodevelopmental disorders (23). In addition, there are some cases of severe acute respiratory syndrome coronavirus 2 (SARS-CoV-2) and catatonia (24). It is hypothesized that the virus spreads to the CNS via axonal transport crossing the blood brain barrier, or induces molecular mimicry triggering NMDA-receptor encephalitis and leading to catatonia (24). The exact mechanism of catatonia is widely complex and not well understood. Nonetheless, this patient demonstrates an example of possible overlap between medical and psychiatric etiologies of catatonia.

Of note, mild hypokalemia was present in all 3 episodes. This was thought to be secondary to poor oral intake and emesis prior to her episodes. In addition, the patient received adequate repletion of her electrolytes without significant change to her psychotic symptoms. Another notable finding was severe vitamin D deficiency during the first psychotic episode (4.7 ng/mL), which was repleted after discharge. Some studies suggest that low vitamin D levels could exacerbate certain symptom domains in patients with a psychotic disorder (25), however, there is no clear evidence that vitamin D deficiency causes psychosis. The patient had a similar psychotic presentation (third episode) despite the lack of a severe vitamin D deficiency (26.8 ng/mL, normal range 30-80 ng/mL).

One limitation of this case report is that the infection source was not identified. The patient had viral symptoms (rhinorrhea, diarrhea, cough) along with objective signs of infection (fever and tachycardia) prior to each discrete psychotic episode described. Inability to identify her infectious source may be due to limitations of our current diagnostic tools. For instance, after the global COVID-19 pandemic, there was an increased number of case studies describing psychotic episodes related to SARS-CoV-2. This allowed the field to characterize a viral strain that is strongly linked to psychosis with a greater hazard ratio compared to influenza and other respiratory tract illnesses (26). Further medical research may be able to identify more viral strains that may directly be linked to psychiatric manifestations.

Another consideration is the lack of elevation of the inflammatory markers assessed. The patient’s medical workup showed normal C-reactive protein (CRP) and erythrocyte sedimentation rate (ESR) levels. CRP levels are commonly associated with infection; however, they are not elevated in all cases, particularly viral infections when compared to bacterial infections (27). Moreover, a study by Lee et al. highlighted possible discrepancies between CRP, procalcitonin, and interleukin-6 (IL-6) in patients with infections, where some people had normal CRP levels but elevated IL-6, indicating that measuring CRP alone might not be reliable in these cases (28). One suggested mechanism behind the viral hypothesis for schizophrenia involves the activation of the inflammatory pathway through cytokines, with studies showing elevated proinflammatory cytokines, particularly serum IL-6 (29). Elevations of complement proteins such as plasma C1q and C4 have also been shown (7). IL-6, complement levels, and other specific proinflammatory markers were not assessed at the time given limited information regarding their clinical utility. Physiologically, CRP’s synthesis occurs in response to IL-6, with IL-6 levels increasing 24 to 48 hours prior to the elevation of CRP levels (30). Therefore, it is possible that the patient’s IL-6 level may have been elevated during these episodes. Furthermore, it is also crucial to note that normal ESR and CRP levels do not exclude the presence of inflammation or infectious processes.

While there have been other case reports linking SARS-CoV-2 and H1N1 influenza virus to psychotic symptoms, our case demonstrates that viral illness manifesting as psychosis applies more broadly to other upper respiratory viruses. This is important when considering further research into mechanism, characterization, prophylaxis, and treatment involving psychotic disorder due to medical illness. Notably, there is a lack of guidelines on the treatment of developing psychotic disorder due to medical illnesses. We have elected to continue maintenance antipsychotic treatment for our patient, even with complete resolution of her psychotic symptoms. This is in effort to prevent future development of psychotic episodes, as repeated psychotic episodes have been shown to be neurotoxic (31). Nonetheless, there are risks and side effects associated with prolonged antipsychotic use, which warrants the need to better characterize these cases and foster a better understanding of pathophysiology and treatment.

Our case details the disease course, extensive medical workup, and selection of multiple psychotropic treatments in a patient with multiple episodes of psychosis, likely due to another medical condition. It highlights the challenges with identifying the diagnosis, completing appropriate workup, and selecting the best management for patients with similar presentations.

## 5 Patient perspective

We are fortunate to have the patient provide her perspective regarding her treatment course and long-term goals. She shares that it is hard to recollect the events during her hospitalizations. Nonetheless, she is confident that her providers do “a great job at treating my condition as my symptoms resolve at discharge”. She emphasizes that her goals are “to prevent symptoms from occurring”. With indefinite antipsychotic treatment, she feels “a little nervous” as it has caused “a considerable amount of weight gain, fatigue, and hypersomnia”. “I am grateful to be well, so I make the best of the treatment I am receiving. However, I am hopeful that at some point either the underlying cause of these episodes will be discovered and treated оr а better medication regimen will be established that does not entail so many adverse side effects.”

## Data Availability

The original contributions presented in the study are included in the article/supplementary material. Further inquiries can be directed to the corresponding author.
